# The First 75 Days of Novel Coronavirus (SARS-CoV-2) Outbreak: Recent Advances, Prevention, and Treatment

**DOI:** 10.3390/ijerph17072323

**Published:** 2020-03-30

**Authors:** Yuxin Yan, Woo In Shin, Yoong Xin Pang, Yang Meng, Jianchen Lai, Chong You, Haitao Zhao, Edward Lester, Tao Wu, Cheng Heng Pang

**Affiliations:** 1 Faculty of Science and Engineering, University of Nottingham Ningbo China, Ningbo 315100, China; 2 Faculty of Science and Engineering, University of Nottingham Malaysia Campus, Selangor 43500, Malaysia; 3 Ningbo New Materials Institute, University of Nottingham, Ningbo 315042, China; 4 Beijing International Center for Mathematical Research, Peking University, Beijing 100871, China; 5 MITMECHE, Massachusetts Institute of Technology, Cambridge, MA 02139, USA; 6 Faculty of Engineering, University of Nottingham, University Park, Nottingham NG7 2RD, UK; 7 Key Laboratory for Carbonaceous Wastes Processing and Process Intensification Research of Zhejiang Province, The University of Nottingham Ningbo China, Ningbo 315100, China

**Keywords:** coronavirus, SARS-CoV-2, COVID-19, coronavirus-infected pneumonia, novel coronavirus pneumonia, zoonotic pathogen

## Abstract

The recent severe acute respiratory syndrome coronavirus 2 (SARS-CoV-2, previously known as 2019-nCoV) outbreak has engulfed an unprepared world amidst a festive season. The zoonotic SARS-CoV-2, believed to have originated from infected bats, is the seventh member of enveloped RNA coronavirus. Specifically, the overall genome sequence of the SARS-CoV-2 is 96.2% identical to that of bat coronavirus termed BatCoV RaTG13. Although the current mortality rate of 2% is significantly lower than that of SARS (9.6%) and Middle East respiratory syndrome (MERS) (35%), SARS-CoV-2 is highly contagious and transmissible from human to human with an incubation period of up to 24 days. Some statistical studies have shown that, on average, one infected patient may lead to a subsequent 5.7 confirmed cases. Since the first reported case of coronavirus disease 2019 (COVID-19) caused by the SARS-CoV-2 on December 1, 2019, in Wuhan, China, there has been a total of 60,412 confirmed cases with 1370 fatalities reported in 25 different countries as of February 13, 2020. The outbreak has led to severe impacts on social health and the economy at various levels. This paper is a review of the significant, continuous global effort that was made to respond to the outbreak in the first 75 days. Although no vaccines have been discovered yet, a series of containment measures have been implemented by various governments, especially in China, in the effort to prevent further outbreak, whilst various medical treatment approaches have been used to successfully treat infected patients. On the basis of current studies, it would appear that the combined antiviral treatment has shown the highest success rate. This review aims to critically summarize the most recent advances in understanding the coronavirus, as well as the strategies in prevention and treatment.

## 1. Introduction

**Figure 1:**
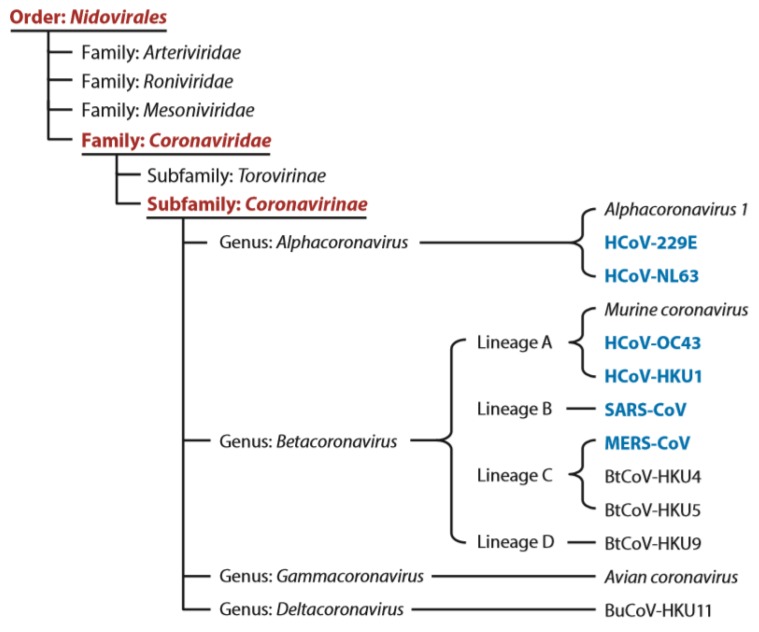
Classification scheme of HCoV and other coronaviruses [6].

**Figure 2:**
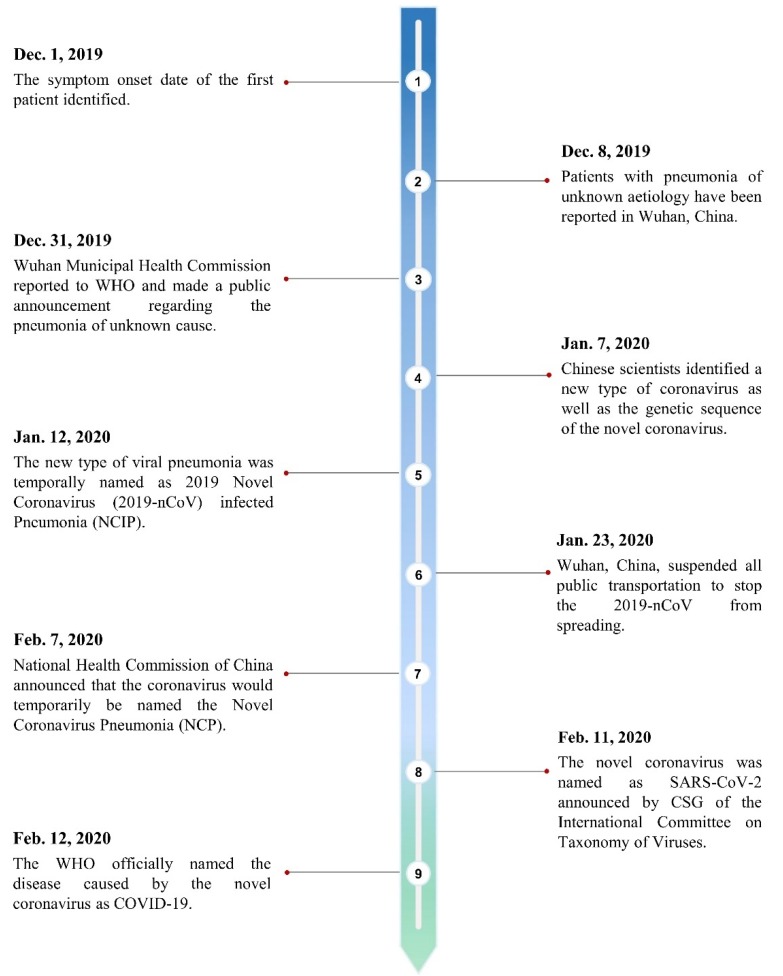
The timeline development of severe acute respiratory syndrome coronavirus 2 (SARS-CoV-2).

Coronavirus, first discovered in the 1960s [1], is a species of enveloped positive-sense RNA virus classified under the order Nidovirales, family Coronaviridae, and the subfamily Coronavirinae (see Figure 1). These are viruses that can infect the cells of certain vertebrate hosts and can be characterized by their club-like spikes protruding from the surface [2]. The subfamily Coronavirinae can be split into four main genera of coronavirus on the basis of their serological and genomic properties, that is, Alphacoronavirus, Betacoronavirus, Gammacoronavirus, and Deltacoronavirus. Betacoronavirus can be further split into four lineages A, B, C, and D (see Figure 1). The recently identified severe acute respiratory syndrome coronavirus 2 (SARS-CoV-2) is now classified into subgenus Sarbecovirus of the genus lineage B Betacoronavirus [3,4]. Due to the widespread availability, large genetic diversity, and frequent recombination of the coronavirus species, in conjunction with the increased time humans spend in the presence of animals, coronaviruses are occasionally able to mutate to infect human hosts [5]. From the list of coronaviruses discovered thus far, there are six species that have been known to infect human hosts (also known as HCoV), causing mild to serious respiratory symptoms depending on the lineage of the coronavirus as well as the immunocompromised nature of the patients [6]. The new coronavirus, initially named the novel coronavirus 2019-nCoV, was first discovered when a cluster of patients reported symptoms of pneumonia of unknown cause to local Chinese health facilities in Wuhan, Hubei Province, in early December 2019 [5]. However, the original source of the novel coronavirus is unclear. On February 11, 2020, the 2019-nCoV was given the name severe acute respiratory syndrome coronavirus 2 (SARS-CoV-2) as announced by the Coronavirus Study Group (CSG) of the International Committee on Taxonomy of Viruses in reference to the 2015 World Health Organization (WHO) naming guidelines [7]. The timeline development of SARS-CoV-2 is shown below (see Figure 2). In the past 20 years, there have been other zoonotic and pathogenic coronaviruses that have led to global or regional outbreaks, including the severe acute respiratory syndrome coronavirus (SARS-CoV) and Middle East respiratory syndrome coronavirus (MERS-CoV). Although not identical, much could still be learned from these previous outbreaks with knowledge and experience incorporated into the current efforts on understanding the SARS-CoV-2 as well as the strategies in prevention and treatment. 

## 2. Epidemiology 

### 2.1. SARS-CoV

The SARS-CoV first emerged as an outbreak of pneumonia in the Guangdong Province of the People’s Republic of China in November 2002. Although it may not have been the first coronavirus to infect humans, it was certainly the first outbreak in modern history of real significance. Empirical antimicrobial treatment for acute community-acquired typical or atypical pneumonia proved to be ineffective in handling the disorder. Upon investigating the pathogens that were causing the pneumonia, it was found that the pathogens have never been identified previously, thus giving this disorder the name severe acute respiratory distress syndrome (SARS) [8]. SARS was caused by a new type of coronavirus, SARS-CoV, and had an incubation period of 4–6 days before patients began developing flu-like symptoms and pneumonia. It was mentioned that by the end of the epidemic in June 2003, SARS had led to over 8000 cases worldwide with a mortality rate of approximately 9.6% [6].

Efforts were then taken to identify the source of the SARS-CoV to prevent future SARS outbreaks. According to some studies on animal samples taken from live-animal markets in Guangdong, it was found that masked palm civets and two other species had already been infected by the SARS-CoV. Because SARS-CoV is zoonotic, this led to the tentative large-scale culling of the masked palm civets. However, further studies later revealed that there were no widespread infections found in wild or farmed civets and clinical symptoms of SARS were only shown to be overt in the civets sold at the markets [9,10], suggesting that civets were highly unlikely to have been the primary/natural reservoir host. The study of Li et al. [9] drew attention to bats and their ability to host several zoonotic viruses whilst rarely displaying any clinical symptoms. In addition, the increased consumption of bats or bat-based products in southern China aroused suspicion that bats might be the primary host of the SARS-CoV. Upon collecting and testing the serum samples and cDNA of 408 bats from nine species, six genera, and three families, Li et al. observed that SARS-CoV tested positive only in different species of horseshoe bats from the Rhinolophus genus [9]. These observations concluded that horseshoe bats were the primary hosts of SARS-CoV, which had subsequently passed it on to other intermediate hosts such as civets and eventually to humans [6]. 

### 2.2. MERS-CoV

MERS-CoV was first discovered in the summer of 2012 in Jeddah, Saudi Arabia, when an unknown coronavirus (at the time) was isolated from the sputum of a patient experiencing acute pneumonia and renal failure. This case could be linked back to April 2012, when healthcare workers in a hospital in Zarqa started developing symptoms of pneumonia. These findings were alarming due to their clinical resemblance to SARS where only a minority of the patients experienced mild diseases, whilst the majority of patients experienced more severe acute respiratory conditions [11]. The MERS-CoV outbreak reached several countries, with the most severe outbreaks found in Saudi Arabia in 2012 and South Korea in 2015, leading to over 2000 cases worldwide with a mortality rate of approximately 35%, mostly consisting of children, the elderly, and people with compromised immune systems [6]. 

Similar to SARS-CoV, MERS-CoV is zoonotic and was believed to have been transmitted to humans via contact with dromedary camels. The dromedary camels are also believed to be an intermediate host and there is no clear answer to the origin of MERS-CoV, except that according to different virus genome analysis, it may have originated from bats that had transmitted the virus to dromedary camels in the distant past. Fortunately, human-to-human transmission of MERS-CoV is fairly uncommon, as close contact needs to be made in order for the virus to pass on to another human host, limiting the spread of the virus [12].

### 2.3. SARS-CoV-2

**Figure 3:**
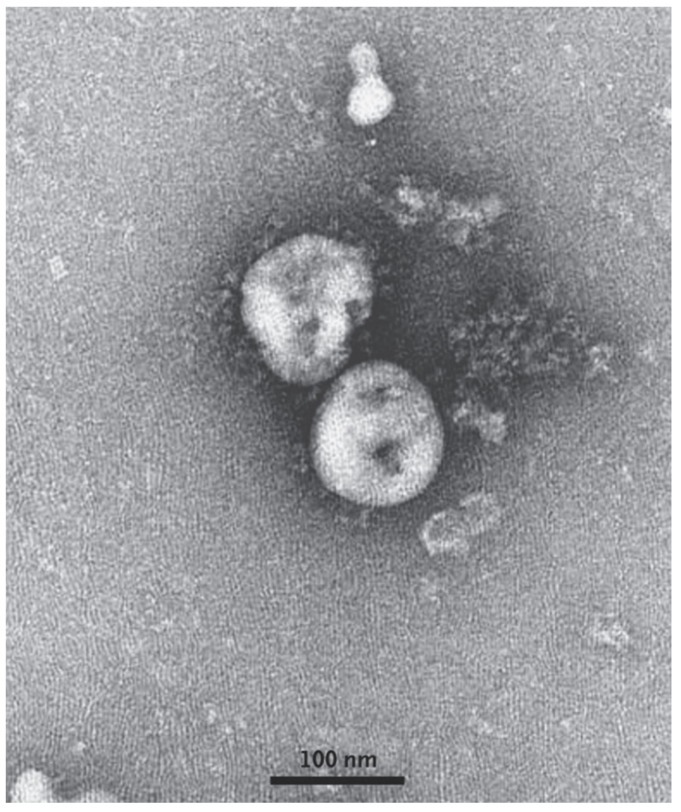
A transmission electron microscope image of SARS-CoV-2 [5].

**Figure 4:**
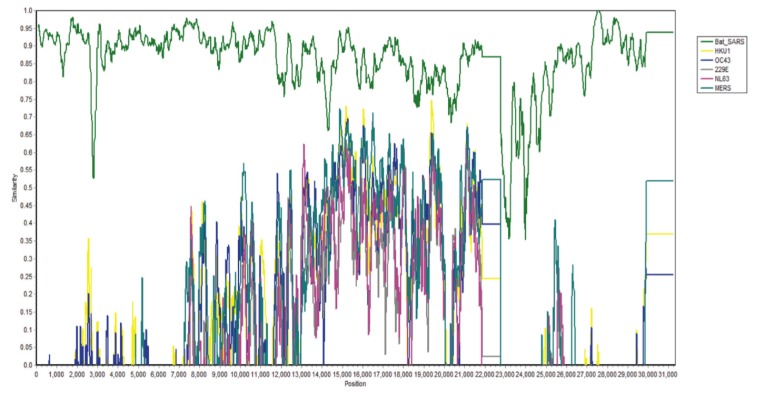
Nucleotide similarity test of SARS-CoV-2 with other known coronaviruses [2].

All 99 patients with confirmed cases of SARS-CoV-2 between January 1 to January 20, 2020, in Wuhan Jinyintan Hospital were studied, and 49% of them were found to have had some form of previous exposure with Huanan Seafood Wholesale Market where live animals were on sale [13]. Full-length genome sequences were released at the early stage of the outbreak. The study revealed that SARS-CoV-2 is 96.2% identical to a bat coronavirus at the whole-genome level and it belongs to the species of SARS-CoV [14]. A transmission electron microscope (TEM) image of SARS-CoV-2 is shown below in Figure 3. According to a test carried out by Phan et al. [2], the nucleotide sequence of SARS-CoV-2 was compared with other types of already known coronaviruses using a pair-wise sequence analysis, and the test results demonstrated an 88% nucleotide similarity to bat-SARS coronavirus (see Figure 4), which was the most similar to SARS-CoV-2 by a significant margin compared to the rest of the known zoonotic coronaviruses with less than 50% nucleotide similarity. Although not definitive, this suggests that the Huanan seafood wholesale market was potentially closely linked to the virus source with other sources, suggesting that bats were the primary reservoir for SARS-CoV-2. 

The SARS-CoV-2 is zoonotic as well as pathogenic, making it one of the coronavirus species that can potentially be transmitted from an animal to a human host. These properties, as well as the long period of time humans and animals spend in close proximity with one another, allow the virus to spread to different hosts at a rapid rate, resulting in global or regional outbreaks [6]. Although there is no current evidence of respiratory disease in infected animals, some (but not all) human hosts develop respiratory symptoms. Such phenomena were also observed in previous SARS-CoV and MERS-CoV outbreaks, which still have no vaccines available. 

SARS-CoV-2, SARS-CoV, and MERS-CoV are classified under the same subfamily, “Coronavirinae”, and are therefore zoonotic, displaying the ability transfer to an intermediate human host. As mentioned previously, the SARS-CoV outbreak occurred where civets played the role of the intermediate host in passing the virus to human hosts in 2002–2003, whilst dromedary camels played the role of the intermediate host passing the virus to humans during the MERS-CoV outbreak from 2012. SARS-CoV had a growth factor of 2–5, whereas MERS-CoV had a growth factor of less than 1 [15]. The intermediate host of SARS-CoV-2 that had passed the virus to humans still remains unclear due to the presence of several animal species at the Huanan Seafood Wholesale Market [16]. 

A full sequence of SARS-CoV-2 has been released by researchers. Phylogenetic analysis revealed that the gene sequence of SARS-CoV-2 is 89% identical to that of bat SARS-like coronavirus ZXC21 (bat-SL-CoVZXC21, accession no. MG772934.1) and ZC45 (MG772933.1), and 82% identical to that of SARS-CoV Tor2 (JX163927), suggesting that SARS-CoV-2 also belongs to betacoronavirus lineage B, but has closer homology to bat-SL-CoVZC45 and bat-SL-CoVZXC21 than SARS-CoV [16]. Structural analysis of the spike (S) protein of this new virus showed that its S protein only binds weakly to the angiotensin-converting enzyme 2 (ACE2) receptor on human cells, whereas the human SARS-CoV exhibits strong affinity to the ACE receptor [17]. Despite this, the binding strength between SARS-CoV-2 and ACE2 is still much higher than the threshold required for virus infection [13,18]. Although this is important in showing the potential for human-to-human transmission, it also shows that binding with ACE2 receptor may not be the only factor affecting virus transmission. In addition, there is increasing evidence that points to human-to-human transmission in hospitals and family gatherings [19,20,21,22,23].

## 3. Clinical Manifestations of SARS-CoV-2

**Figure 5:**
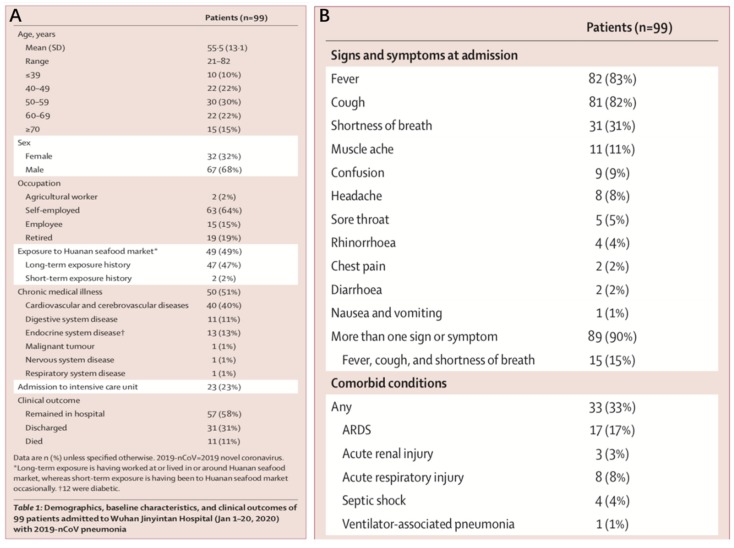
**(A**) Summary of patients’ background; (**B**) summary of patients’ clinical symptoms [13].

From a study conducted by Chen et al. [13], medical reports of 99 patients up to January 25, 2020, were examined. The age of the patients ranged from 21 to 82 years old, with a mean age of 55.5. Approximately 32% of the patients were female and 68% male. From this sample of patients, about half of them were suffering from other underlying cardiovascular and cerebrovascular diseases. It would appear that older individuals and/or individuals with compromised immune system are at a greater risk of infection. In addition, although preliminary, it would seem as though there is a link to the sex of patients. The lower number of female patients, as observed in this particular group of patients, could potentially be attributed to their natural immune system protection from X chromosomes and sex hormones (see Figure 5A) [13], which are known to play a key role in innate and adaptive immunity [24] and thus less susceptible to various infections. That being said, further observations and studies are needed to confirm this relationship. The patients also displayed various clinical symptoms, such as fever (83%), cough (82%), shortness of breath (31%), muscle ache (11%), confusion (9%), headache (8%), sore throat (5%), rhinorrhea (4%), chest pain (2%), diarrhea (2%), and nausea and vomiting (1%) (see Figure 5B). Approximately 90% of the patients were observed to have more than one of the symptoms, whereas 15% experienced fever, cough, and shortness of breath simultaneously. Laboratory tests displayed an overall fall in the value of lymphocytes, suggesting that SARS-CoV-2 might mainly target lymphocytes, specifically T lymphocytes, by spreading through the respiratory mucosa, triggering immune responses and negative changes in the immune system. This would potentially cause acute respiratory distress syndrome (ARDS) and septic shock in some patients, leading to eventual organ failure [13].

**Figure 6:**
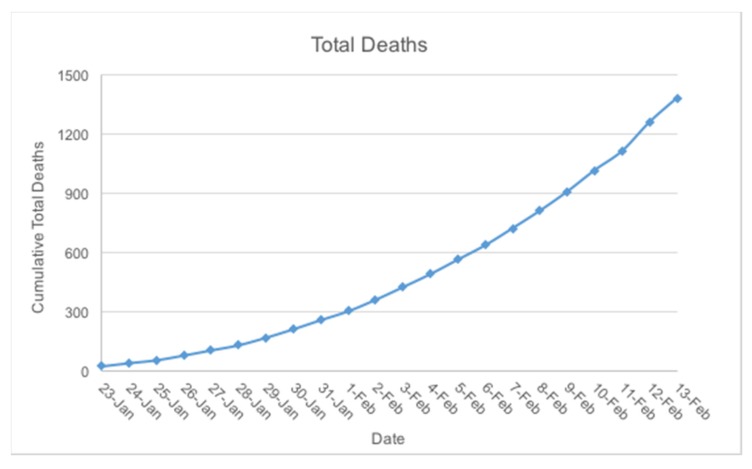
Trend of death rate as of February 13, 2020 [25,26,27].

As of February 13, 2020, a total of 1370 deaths from coronavirus disease 2019 (COVID-19) have been reported with 60,412 confirmed cases in 25 different countries. According to a live update, the number of deaths resulting from the COVID-19 is shown to be rising with an overall daily growth factor of over 1, which suggests an exponential growth in the number of deaths January 23, 2020, to February 13, 2020 (see Figure 6) [25,26,27]. According to a study on medical reports carried out by Zhao et al., the total number of unreported cases from January 1 to January 15, 2020, was estimated to be 469, with a growth factor of 2.56 [28].

As the COVID-19 outbreak is taking place in the middle of the flu season, symptoms in the early stages could have been ignored and gone unreported due to the similarity with the symptoms of common influenza, allowing the infection to progress to its later, more severe stages. The mortality rate of the COVID-19 has been estimated to be 2% thus far, but this may change due to the lack of information obscuring the full picture of this outbreak. After the first 75 days, the WHO had not announced this as a pandemic but instead a “public health emergency of international concern” [29].

**Figure 7:**
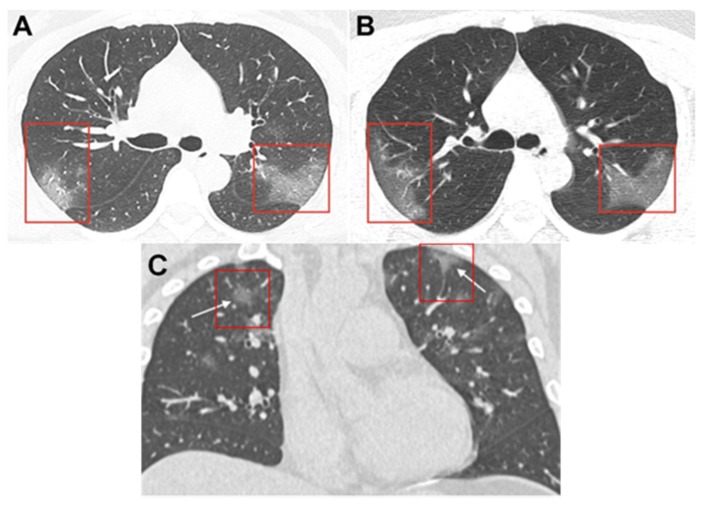
Unenhanced computerized tomography (CT) images of infected lungs of a 33-year-old woman. (**A**) Image showing multiple ground-glass opacities in bilateral lungs [33]; (**B**) image obtained 3 days after follow-up showing progressive ground-glass opacities in the posterior segment of the right upper lobe and apical posterior segment of left superior lobe [33]; (**C**) coronal thin-section non-contrast CT image of infected lungs of a 36-year-old male showing ground-glass opacities with a rounded morphology in both upper lobes [34].

The study of Zhong et al. showed that acute respiratory distress (ARD) caused by SARS-CoV-2 was diagnosed amongst the whole spectrum of age, whereas 0.9% of patients were below the age of 15 [30]. The median age was 47, and 58.10% were males. The common clinical symptoms were fever (87.9%) and cough (67.7%). Ground-glass opacity (50.0%) and bilateral patchy shadowing (46.0%) were typically found on chest computerized tomography (CT) scans. Figure 7 shows ground-glass opacity in the infected lungs of both male and female COVID-19 patients of similar age range. The median incubation period is 3.0 days (with incubation period ranging from 0 to 24 days). COVID-19 has a relatively lower fatality rate than SARS-CoV and MERS-CoV. It was found that the mortality rate of 1.4%, based on the larger sample size studied, is lower than that reported previously [13,30,31,32]. 

## 4. Transmission

### 4.1. Human-to-Human Transmission

As mentioned in Section 2.3, detailed genomic and structural analysis of SARS-CoV-2 showed similarities to the SARS-CoV and close derivative of other zoonotic bat coronaviruses such as bat-SL-CoVZC45 and bat-SL-CoVZXC21. However, according to structural analysis of the spike (S) protein in SARS-CoV-2, the S protein shows that it can only form weak bonds to the ACE2 receptors in human cells, suggesting that the virus may not transmit readily between human hosts, reducing its contagiousness and reproduction number [28]. However, 1099 patients with laboratory-confirmed COVID-19 were studied by Zhong et al., and only 1.18% of them had direct contact with wildlife, thus suggesting a high potential of human-to-human transmission. The routes of transmission might have accelerated the spread of SARS-CoV-2. Apart from respiratory droplets and direct contact, which serve as the conventional routes of transmission of SARS-CoV, MERS-CoV, and highly pathogenic influenza [32,35,36], fomite transmission was found to be a potential reason for the rapid transmission of SARS-CoV-2 [30]. 

An observation on January 20, 2020, showed that the infection had sharply increased in the span of a few days, reaching a total of 440 confirmed cases, of which 15 were medical workers associated with previous patients hosting the SARS-CoV-2 [17]. In another study carried out by Chan et al., six family members who travelled to Wuhan from Shenzhen in Guangdong Province, China, were monitored. Five of them were tested positive for the COVID-19, despite not having come in contact with any of the markets or animals in Wuhan where the outbreak is believed—although not definitively—to have started. An additional family member was also reported to have contracted the disease several days after coming in contact with four of the family members. This observation further reinforces the fact that SARS-CoV-2 can be transmitted from a human host to another human host [37]. This also suggests that SARS-CoV-2 may be able to undergo mutation and adapt to infecting human hosts more easily than before, creating the urgency to find methods to contain the mutation. A method of predicting whether or not these mutations will lead to further infectivity in the future is available but has yet to be conducted and validated [17]. 

According to the WHO, the current basic reproduction number (R_0_) is estimated to be within 1.4-2.5, exceeding that of the SARS-CoV in 2012. This means that each human host of the SARS-CoV-2, on average, spreads the virus to 1.4-2.5 more hosts [15]. Thus far, all human-to-human infections have been classified as second generation transmission, and there have yet to be any known cases of third generation transmission, which is a human infecting another human who was previously infected by another human. If this were to occur, it could spell out an increase in the reproduction number and the possibility of the virus transmitting [38]. Apart from China, human-to-human transmission has been reported in other countries such as Germany, Japan, Thailand, and the United States [39]. In a paper published in the New England Journal of Medicine (NEJM), it was reported that the first four cases of the COVID-19 in Germany were associated with a businesswoman from Shanghai who travelled to a company in Munich on January 20 and 21. The four people whom she met soon started experiencing clinical symptoms, and it was later reported that the businesswoman herself was not experiencing symptoms at the time, leading experts to believe that asymptomatic patients are able to transmit the SARS-CoV-2 as well. Although it was later found out that the businesswoman had suppressed the symptoms with paracetamol [40], there is yet enough evidence to rule out the potential of asymptomatic transmission.

As discussed in Section 4.1, the number of people that one infected person can spread to is defined by the reproduction number, R_0_. It is important to understand that R_0_ is an average of the number of people one infected host can infect and can therefore change according to different scenarios, such as how the virus is spread and for how long an infected host can spread the virus before recovering. This makes the R_0_ value different for every location and patterns are still emerging in each country [41]. 

According to a study conducted by Imai et al., on the basis of the frequency of international travel from Wuhan up to January 18, 2020, the average R_0_ value in Wuhan was found to be 2.6, with an uncertainty range of 1.5–3.5. This shows that an average infected person in Wuhan could potentially spread the virus to roughly 2.6 other patients [42]. Another study carried out by Zhao et al. [28] reported that the R_0_ was estimated to range within 2.49 to 2.63 on the basis of the confirmed cases reported in Wuhan between January 1 and January 15, 2020. In the works of Shan et al., the R_0_ in Wuhan was estimated to be within the range of 4.50 to 4.92 during the initial period of the epidemic on December 12, 2019, and dropped to 1.99–2.18 as of January 22, 2020. This shows that the current efforts of quarantine, travel restrictions, contact tracing, and other interventions are effective in controlling the outbreak. That being said, the number of infected individuals in China are expected to reach its peak in early March [43]. As of the current moment, all sources display a R_0_ value of more than 1; hence, an exponential growth in the number of cases can be expected.

### 4.2. Transmission Model

Researchers are exploring a wide range of resources to examine the transmission dynamics of SARS-CoV-2 for better prevention and control of the current outbreak. The origin and diffusion epicentre of SARS-CoV-2 were investigated via phylogeny and maximum-likelihood mapping analyses. On the basis of the time-scale phylogenetic analysis of the virus genome sequences, the potential large “first generation” human-to-human virus transmission is estimated to have occurred on November 9, 2019 [44]. Similarly, phylogeographic analysis of the two geographical locations with earliest confirmed cases reported (primarily Wuhan, China, and Bangkok, Thailand [45]) revealed that most viral transmissions between epidemiologically linked cities potentially originated from Wuhan, with a posterior probability of 0.98 [44], rather than from Bangkok. However, more evidence and studies will be needed to confirm the origin. 

With the alarming increase in the number of confirmed cases, various researchers began their studies by estimating the R_0_ of the COVID-19. R_0_ is an epidemiologic matrix used to describe the transmissibility of infectious agent. Various studies [22,28,46,47,48,49,50] indicated the number of expected cases directly generated by a single infected case is within the range of 0.8–5.7. As the aforementioned studies approximated the virus incubation period at 5.2–12.5 days, the expected epidemic doubling time of COVID-19 could be in the range of 2.9–8.4 days. The probability of outbreak in locations outside of Wuhan is estimated on the potential individual-level variation in transmission, in which the increase in variation will result in lower possibility of an outbreak, whereas the increase in transmission homogeneity will most likely establish an outbreak in a new location. Researchers anticipated 50% possibility [47] of an outbreak occurring in a new location by assuming a SARS-like variation and a Wuhan-like transmission in the study model. 

The epidemic dynamic network values are obtained via estimation methods such as sequential Markov Chain Monte Carlo algorithm [22,47], Poisson and log-likelihood regression [22,28,47,49,51], and Euler–Maruyama algorithm [47], with uncertainties and approximations quantified by Bayesian analysis [46,49,51]. Most of the above-mentioned studies simulated the Wuhan epidemic dynamic network by utilizing a deterministic susceptible-exposed-infected-recovered (SEIR) model that categorizes the population into four states: susceptible individuals (S), asymptomatic individuals during the incubation period (E), confirmed infectious individuals (I), and recovered individuals (R). A SEIRDC model enhanced from the standard SEIR model by introducing dead and auxiliary variable (DC) [50] is also used for COVID-19 investigation. A geological-stratified debiasing approach is incorporated into the SEIRDC model to include the latent infection ratio (L&I) amongst the people traveling from Wuhan to other destinations in mainland China to improve the overall epidemiological estimation of the coronavirus. 

Studies on the infectivity of SARS-CoV-2 include analyzing the genomic structure of SARS-CoV-2 [17], examining the interactions between human and SARS-CoV-2 receptors [52] and establishing a reservoir–people transmission network model [53]. The analysis of the major structural proteins including the spike (S), membrane (M), envelop (E), and nucleic capsid (N) proteins of SARS-CoV-2 showed that the coronavirus exhibits a high degree of variation from the human SARS virus and should be categorized as a novel type of bat coronavirus [17]. As the transmission of coronaviruses to human is, in part, driven by the interaction of spike proteins (S protein) with human receptors ACE2, researchers have utilized the Monte Carlo algorithm to simulate the docking and binding processes of SARS-CoV-2 S protein to ACE2 [52]. Simulation results showed that the binding affinity of SARS-CoV-2 is 73% similar to SARS-CoV, suggesting its S protein has been encoded by its genome to process human receptor-binding ability and thus it is highly possible that it can drive human–human transmission. On the other hand, the transmissibility from the infection source to human is simulated on the basis of a reservoir–human transmission network model. The matrix employs the SEIR model for both infected source and human population. As the R_0_ varies with every updated input into the matrix, the model suggests that if R_0_ > 1, the outbreak will occur; if R_0_ < 1, the outbreak will be terminated [53]. 

## 5. Infection Prevention and Control Methods

### 5.1. Recommendation by the WHO

In order to reduce the risk of contracting the SARS-CoV-2, the WHO has recommended several hygiene and personal practices to adopt. The first recommendation is the regular washing of hands with soap and water—a simple, yet effective, action to mechanically remove pathogens from human skin. Washing hands for 15 seconds has been shown to reduce the pathogen count by roughly 90%, whereas 30 seconds removes up to 99.9% [54]. Thoroughly washing hands has been shown to reduce respiratory illnesses in the general population by 16–21%.

It has also been highly recommended that hands be cleaned with alcohol-based hand rubs consisting of ethyl alcohol (ethanol), isopropyl alcohol (isopropanol, propan-2-ol), and *n*-propanol. Alcohols exhibit rapid broad-spectrum antimicrobial activity against vegetative bacteria, viruses, and fungi [55]. Optimal antimicrobial activity occurs at an alcohol concentration between 60% and 90%, whereas activity is significantly lower at concentrations below 50%. When alcohol is used, the cytoplasmic membrane of the virus particles would be damaged due to the rapid denaturation of proteins making up the membrane. This causes the viral nucleic acid to be released into the environment, followed by capsid destruction and viral inactivation [56].

The WHO also recommends practicing respiratory hygiene, which includes, but is not limited to, covering one’s own mouth when coughing or sneezing. Coronavirus is known to spread through larger droplets that are expelled during coughing or sneezing [57]. The WHO also recommends maintaining suitable social distance of at least 3 feet from surrounding people and to particularly avoid any form of physical contact or activity that might encourage the projection and transfer of small droplets that could potentially carry the virus [55].

The WHO also recommends wearing personal protective equipment, specifically surgical face masks or N95 respirators, as an infection-control strategy to protect the wearer from liquid and airborne particles [58]. A surgical face mask is effective at blocking out sprays of larger droplets, preventing them from reaching the mouth and nose. However, it is unable to completely block out smaller droplets in the air that might have been transmitted from coughs, sneezes, or certain medical procedures. The loose contact between the face and surface of the mask provides some exposure for droplets to make their way through and enter the mouth or nose. An N95 respirator, on the other hand, when worn correctly, blocks out at least 95% of small (0.3 micron) particles, making it a better option for stemming the outbreak of the new coronavirus [59]. However, N95 respirators need to be fit-tested to ensure effective protection and prevention.

### 5.2. Recommendations by Various Governments and Agencies

Governments in various countries have also raised awareness amongst the public and have provided similar recommendations for prevention and containment of the SARS-CoV-2. Justifications for each of the recommendation are similar to the ones described in Section 5.1. The recommendations by various countries and agencies are shown below in Table 1. 

## 6. Therapy

Within a human host, the coronavirus is known to spread through the respiratory mucus membrane to infect other cells within the body, whilst inducing a cytokine storm and generating a sequence of immune responses [13]. Specifically, the SARS-CoV-2 is known to affect the peripheral blood cells and other immune cells, particularly the lymphocytes. Many COVID-19 patients have shown reduced number of lymphocytes and, in some cases, lymphopenia, thus indicating the detrimental effects of the SARS-CoV-2 on immune cells and system. This is similar to SARS-CoV, where lymphocytes, mainly the T lymphocytes, are adversely affected. Damaged T lymphocytes are possibly one of the main factors resulting in complications in COVID-19 patients [13].

Most COVID-19 patients develop symptoms including fever, dry cough, dyspnea, and bilateral ground-glass opacities, as evident on chest computerized tomography (CT) scans [31], whereas others have also shown symptoms such as headache, muscle ache, chest pain, confusion, and diarrhea [13]. Further health complications include ARDS, RNAaemia, acute cardiac injury, and secondary infection [30]. That being said, respiratory damage is the leading cause of death in patients diagnosed with COVID-19.

Since the outbreak, a series of medical interventions have been administered to patients infected with SARS-CoV-2. However, many of these treatments were not specifically designed to treat COVID-19, but rather to treat diseases with similar symptoms to COVID-19 in the past. Given the limited knowledge on SARS-CoV-2 and COVID-19, the efficacy, mechanism, and direct effect of these treatments on this rather unknown viral respiratory infection are not yet fully understood. Clearly, more detailed, systematic, and thorough investigations, both in vitro and in vivo, are needed. As of now, some medical treatments have been proposed (Section 6.3, Section 6.4, Section 6.5, Section 6.6 and Section 6.7), and some others administered (Section 6.1 and Section 6.2), on the basis of theoretical studies and previous similar experience.

### 6.1. Antiviral Therapy 

Oxygen therapy, mechanical ventilation, intravenous antibiotics, and oseltamivir therapy were initiated in 38.0%, 6.1%, 57.5%, and 35.8% of patients, respectively [30]. Antiviral therapy can be applied to patients when a viral infection has already been established in a host and needs to be controlled [67]. One of the prescribed antiviral medications for patients suffering from COVID-19 is oseltamivir, which is normally used for treating influenza in adults, children, and infants older than 2 weeks of age who have experienced clinical symptoms for no more than 2 days. Oseltamivir is classified under a class of medicines known as neuraminidase inhibitors that work by stopping the spread of the virus throughout the body, shortening the time in which the flu symptoms last [68]. Neuraminidase inhibitors promote the release of virus from infected cells and facilitate viral movement within the respiratory tract. Virions remain attached to the membrane of infected cells in the presence of neuraminidase inhibitors and get stuck in respiratory secretions [69].

Another type of antiviral medication used for SARS-CoV-2 patients is ganciclovir [13], normally used to treat cytomegalovirus (CMV) retinitis in patients with compromised immune system and at risks of inhibiting virus replication [70]. Ganciclovir is first converted to its active form, monophosphate, whilst being catalyzed by a virus-coded cellular enzyme known as thymidine kinase (TK) [71]. Monophosphate is then converted into ganciclovir triphosphate by cellular enzymes which, competitively, inhibits dATP, leading to the formation of ‘faulty’ DNA by replacing many of the adenosine bases in the DNA strand and preventing DNA synthesis [71]. 

Remdesivir, a broad-spectrum antiviral nucleotide prodrug, has shown promising antiviral activity against various coronaviruses in vitro and is accessed for activity against SARS-CoV-2 [72,73,74]. Remdesivir inhibits viral replication by initializing efficient metabolic conversion in cells and tissues to activate nucleoside triphosphate which, in turn, deactivates viral RNA polymerases [75]. Remdesivir has been shown to have a diminishing effect on the pathological features of acute lung injury (ALI), a common clinical feature observed in COVID-19 patients. Previously, in vivo studies have shown that lung injury scoring was significantly reduced in MERS-CoV-infected mice treated with remdesivir [72,73,74]. Sections of the lung tissue appeared to have a lower concentration of debris and inflammatory cell deposition, thus facilitating a more efficient gas exchange in the lungs as compared to those of untreated mice with ALI [72,73,74]. Apart from lung injuries, some COVID-19 patients have also developed secondary infection and septic shock, a widespread infection throughout the body that would result in multiple organ failures. Non-human primate tissue distribution studies have shown that remdesivir can effectively penetrate and distribute into immune-privileged sites, including the eyes, genital tracts, and some extent of the brain, which could be a persistent reservoir for viruses [75]. This would allow remdesivir to provide a wider coverage of antiviral effect to different organs in the body and thus reduces the possibility of virus accumulation in immune privileged sites. This indicates the potential of remdesivir as an antiviral drug to treat COVID-19 patients.

In the past, lopinavir and ritonavir have been prescribed to patients suffering from SARS-CoV and MERS-CoV to reduce clinical symptoms. A combination of lopinavir and ritonavir have been administered to patients suffering from COVID-19 [31]. KALETRA, a co-formulation of lopinavir and ritonavir, is also tested for activity against SARS-CoV-2 [72]. New proteins such as structural proteins and DNA-manufacturing enzymes are made by viral protease during the production of viruses [76]. Lopinavir prevents the cleavage of group-specific antigen-polymerase (Gag-Pol) polyprotein in virus, inhibiting the protease action and resulting in the production of defective, immature, and non-infectious viral particles. By co-formulating in KALETRA, ritonavir increases the half-life and plasma level of lopinavir by inhibiting the oxidation caused by cytochrome P450 [77].

Other drugs such as ribavirin, penciclovir, nitazoxanide, nafamostat, chloroquine, and favipiravir are proposed and tested in vitro [78]. Amongst the mentioned drugs, ribavirin, penciclovir, and favipiravir require high concentration of nucleoside analogs to reduce the viral infection and are considered less effective. Nafamostat and nitazoxanide, capable of preventing membrane fusion and acting as an antiprotozoal agent, respectively, exhibit inhibitive activity against COVID-19. As for chloroquine, its ability to interfere with the glycosylation of cellular receptors of SARS-CoV and to inhibit virus infection by increasing endosomal pH required for virus fusion has recently been reported and hence is a potential broad-spectrum antiviral drug. Therefore, nafamostat, nitazoxanide, and chloroquine are of high potential and are recommended for evaluation in vivo for patients suffering from COVID-19.

Another effective antiviral strategy is to disrupt the AP2-associated protein kinase 1 (AAK1) that regulates viruses’ passage to cell via endocytosis. Amongst the 47 AAK1 inhibitors approved for medical use that have been studied [79], oncology drugs such as sunitinib and erlotinib have shown effective viral infection inhibition. However, the side effects that coexist with its usage could not support a safe therapy for sick and infected patients. An alternative high-affinity AAK1-binding drug, baricitinib, is found to bind with another regulator of endocytosis, the cyclin G-associated kinase. Hence, baricitinib appears to be a potential kinase-inhibiting drug for the COVID-19. 

Drugs that target the viral protease, which is the highly conserved key enzyme for coronavirus replication, are commonly capable of preventing proliferation of the virus. Prulifloxacin bictegravir, nelfinavir, and tegobuvi are protease-targeting drugs that interrupt the dimer formation of the 5n5o protease [80]. Valrubicin, icatibant, bepotastine, epirubicin, epoprostenol, vapreotide, aprepitant, and caspofungin are examined via virtual docking and appeared to exhibit high hydrogen bond binding with the main protease (Mpro), in succession interfering with the functions of SARS-CoV-2 Mpro [81]. On the basis of the results, the antiviral drugs proposed above could serve as potential candidates for protease-targeting treatment developed to treat COVID-19. 

As for the current studies, a limited number of detailed investigations have been carried out specifically on SARS-CoV-2 and COVID-19. Wang et al. [78] examined the effects of a series of antiviral drugs, including ribavirin, penciclovir, nitazoxanide, nafamostat, favipiravir, chloroquine, and remdesivir, in vitro over SARS-CoV-2. Some promising results have shown that the EC_90_ value of remdesivir against SARS-CoV-2 in Vero E6 cells is 1.76μM, suggesting its working concentration is likely to be achieved in nonhuman primate models, and remdesivir was found to inhibit virus infection efficiently in a human cell line (human liver cancer Huh-7 cells), which is sensitive to 2019-nCoV [78]. On the contrary, other antiviral drugs, such as oseltamivir and KALETRA, have been tested over H5N1 and MERS-CoV, rather than SARS-CoV-2 [74,82].

### 6.2. Antibiotic Therapy

Antibiotics have been prescribed to COVID-19 patients with low immune function, namely, the elderly and medically fragile patients. The administration of antibiotics prevents bacterial co-infections whilst strengthening the immune support, leading to better chance of recovery. Antibiotic treatments have demonstrated positive therapeutic outcomes in various clinical studies [13,30,31]. Specifically, cephalosporins, carbapenems, and quinolones are antibiotics that have been given to COVID-19 patients, either in single or multi-antibiotic treatment, in parallel with antiviral agents. The epidemiological study reported that the combined treatment has seen some success in treating COVID-19, with patients reportedly recovering and then being discharged from hospitals [13]. 

Cephalosporin, the bactericidal beta-lactam antibiotic, disrupts bacterial cell synthesis by inhibiting the enzyme actions in cell wall of susceptible bacteria [83]. Cephalosporin binds with penicillin-binding protein (PBP), the bacterial enzyme responsible for synthesizing the peptidoglycan, thus affecting the structural integrity of the bacterial cell wall [84]. The inhibition of cell wall formation, an integral part of bacterial structure and cell survival, results in the bacteria being susceptible to surrounding attacks and eventually leads to cell death. 

Similar to cephalosporin, carbapenem disrupts the cell wall formation of the bacteria by inhibiting the active sites of PBPs. Additionally, carbapenem obstructs peptidase reactions and peptide cross-linking by binding to the peptidase domains [85]. As the activity of PBP is inhibited whilst the autolysis process is developing, during cell wall formation, the peptidoglycan layer weakens, followed by bacteria bursting due to osmotic pressure. 

Quinolones exhibit antimicrobial effects by converting the enzymes that are essential for bacterial DNA replication and transcription, namely, gyrase and topoisomerase IV, into toxic enzymes that would fragment the bacterial chromosomes [86]. Quinolones bind at the enzyme–DNA interface, interacting with the protein whilst intercalating into the DNA at both cleaved scissile bonds. As the drug-stabilized gyrase– or topoisomerase IV–DNA cleavage complexes collide with transcription complexes during replication, these complexes are converted into permanent chromosomal breaks. The excessive DNA breakage disrupts cell function, resulting in bacterial death.

### 6.3. Fusion Proteins

By examining the function of ACE2 for coronavirus binding, a proposed ACE2-fused protein is hypothesized to exert neutralization potential for coronavirus, especially SARS-CoV-2 [87]. A fusion protein (ACE2-Ig) constructed out of extracellular domain of human ACE2 receptors, linked to the Fc domain of human immunoglobulin IgG1, was tested for both SARS-CoV and SARS-CoV-2. The half maximal inhibitory concentration (IC_50_) tested in vitro to neutralize SARS-CoV and SARS-CoV-2 were 0.8 and 0.1 μg/ml. The cross-reactivity and high binding affinity of the fusion protein to the receptor-binding domain (RBD) of SARS-CoV and SARS-CoV-2 suggest that it is a potential treatment strategy for COVID-19.

### 6.4. mRNA Vaccines

A coronavirus vaccine that converts SARS-CoV-2 viral spike protein sequences into messenger RNA (mRNA) [88] is under development. In vitro transcribed mRNA is engineered to resemble a fully processed mature mRNA molecule and could be degraded quickly by extracellular RNases. The cellular translation machinery takes place once the mRNA enters the cytosol and the fully functional proteins are produced after the post-protein-translational modifications [89]. The vaccine is anticipated to initiate immune cells in the lymph to process the coded mRNA and, in turn, produce proteins in a manner where other immune cells recognize and begin mounting relevant responses against an actual viral infection [90].

### 6.5. DNA Vaccines 

DNA vaccine encoding the first 725 amino acids (S1) of MERS-CoV spike (S) protein have shown to increase the secretion of interferon gamma (IFN-γ) and other cytokines by antigen-specific CD4+ (often known as helper cells) and CD8+ (often known as cytotoxic or killer cells) T cells, inducing protection against MERS-CoV [91]. Progressing from the development of MERS-CoV vaccines, DNA vaccines for COVID-19 that rely on spike proteins have also been closely studied [88]. The proposed vaccine with targeted viral DNA sequences is expected to prepare the immune system and launch a strong response against the pathogen [92].

### 6.6. Cellular Therapy

COVID-19 is known to be associated with overt inflammatory responses [13,31]. The use of mesenchymal stromal cells obtained from allogeneic donors in cellular therapy facilitates the regeneration of damaged cells and thus is effective in reducing non-productive inflammation in patients [93]. Studies have shown that autologous hematopoietic stem-cell transplant could result in short-term expansion and isolation of antivirus-directed T cells for cytomegalovirus infection treatment. Hence, the expansion of anti-SARS-CoV-2-specific T cells is believed to be a potential adjunct treatment for patients diagnosed with COVID-19 [94]. 

### 6.7. Epitope Targeting for Vaccine Design 

Depicting the common epitopes is essential to design a universally potent sub-unit vaccine. The epitopes that are commonly recognized by all dominant human leukocyte antigen–DR isotype (HLA-DR, a major histocompatibility complex class II cell surface glycoprotein) alleles were investigated [95]. Eight immunodominant HLA-DR epitopes distributed across the spike, envelope, and membrane proteins exhibited high affinity and suggested the potential effective antiviral T cell and antibody responses against SARS-CoV-2. Similarly, by screening SARS-CoV-derived B cell epitopes, 61 epitope sequences from the structural proteins comprised no mutation and had an identical match to the existing SARS-CoV-2 protein sequences [96]. Therefore, these epitopes have great potential in eliciting an effective cross-reactive response against SARS-CoV-2. 

### 6.8. Cases of Successful Treatment

According to existing case studies, there have been numerous cases of patients recovering from COVID-19. As of February 13, 2020, a total of 6271 COVID-19 patients in various countries had received relevant medical treatments and were discharged from hospitals. This was a promising start with the numbers still on an increasing trend. A 71-year-old Chinese female COVID-19 patient, under a combined treatment of oseltamivir, lopinavir, and ritonavir, was tested negative for the viral infection for 48 hours consecutively and was discharged [97]. On February 3, Cable News Network (CNN) reported that the first U.S. patient in Washington, a man in his 30s, was treated and discharged from hospital. He remained under isolation at home whilst being closely monitored by the Snohomish Health District [98]. On February 4, a 35-year-old man from Wuhan, China, was discharged from a hospital in Singapore. Prior to his discharge, he was tested negative for COVID-19 in 3 continuous days [99]. On the same day, a 4-year-old girl from Malaysia was discharged from a hospital in Langkawi [100]. On February 7, two patients in their 60s from suburban Chicago were discharged from hospital, but remained in self-quarantine and observation [101]. On February 8, a 40-year-old man from Wuhan was discharged from Permai Hospital in Johor Bahru, Malaysia, after being treated with KALETRA for 8 days [102]. 

In China, 28 of the 41 patients in Wuhan Jin Yin-tan Hospital were discharged as of January 22, 2020 [31]. Similarly, an epidemiological study of 99 COVID-19 patients in Wuhan reported that 31 patients have recovered and were discharged as of January 25, 2020 [13]. To date (February 13, 2020), a total of 2016 patients have reported recovery from COVID-19 in Wuhan alone (35,991 confirmed cases). Hospital discharge rates vary between 5% and 68% in different hospitals in Wuhan, whereas the average recovery rate recorded across China is 10.5%. 

In another report, the clinical characteristics of pregnant patients in Zhongnan Hospital of Wuhan University were studied in order to investigate the potential of intrauterine vertical transmission of COVID-19. It was found that the clinical characteristics of pregnant COVID-19 patients are similar to non-pregnant adult patients. Amongst the nine pregnant patients, all of them were provided with antibiotic treatment and nasal cannula oxygen support, whilst 67% of them were given antiviral drugs. As of February 4, 2020, nine live births were recorded with no evidence of vertical transmission in late pregnancy of COVID-19 patients [103]. 

### 6.9. Recommendations Based on Present Knowledge Gathered in the First 75 Days of COVID-19 Outbreak

Current medical strategies administered to patients diagnosed with COVID-19 mainly include the use of antiviral drugs, antibiotic agents, and, in some severe cases, oxygen therapy and renal replacement therapy as well. Depending on the severity of symptoms, either single or a combination of treatment is given to COVID-19 patients. Antiviral treatment, primarily using oseltamivir, ganciclovir, and KALETRA (a combination of lopinavir and ritonavir), is prescribed to treat the ARD and to minimize the potential of septic shock. In parallel, most patients are also given either single or a combined treatment of antibiotic agents, namely, cephalosporin, quinolone, and carbapenem to prevent secondary infection and to strengthen the immune system. In more severe cases, oxygen therapy and renal replacement therapy are provided as supportive treatment to maintain the functions of vital organs throughout treatment and recovery period. 

Oxygen therapies, including nasal cannula, high-flow nasal cannula, invasive mechanical ventilation, or extracorporeal membrane oxygenation (ECMO), are given to COVID-19 patients suffering from ARDS and oxygen deficiency due to impaired lung functions to ensure adequate ventilation and healthy concentration of oxygen throughout the body. The oxygen therapy eases symptoms of hypoxemia and prevents further organ damage. Renal replacement therapy replaces nonendocrine kidney function in patients with renal failure. Continuous renal replacement therapy (CRRT) is prescribed to COVID-19 patients with acute renal failure to reduce solute imbalance and toxicity overload that could result in multi-organ failure.

On the basis of current studies, it would appear that a combination of antiviral, antibiotic, and supportive treatments potentially provide a higher success rate in treating COVID-19 patients, particularly those with moderate to severe ARDS. 

From an epidemiological study of 41 COVID-19 patients in Wuhan [31], 93% of the patients were prescribed antiviral oseltamivir. In addition, 100% of the patients received antibiotics treatment, 66% of patients were on oxygen therapy, and 7% of patients were given CRRT. The discharge rate from hospitals was as high as 68% [31]. Similar combined treatment has reported promising results in a collective study involving 1099 patients in 552 hospitals across 31 provinces in mainland China [30]. In another epidemiological study involving 99 COVID-19 patients in Wuhan [13], a combination of oseltamivir, ganciclovir, and KALETRA was prescribed to 76% of the patients. A total of 25% of the patients were provided with single antibiotic treatment, whilst 45% of the patients received multi-antibiotic treatments. Of the patients in this study, 76% and 9% were on oxygen therapy and CRRT, respectively. Eventually, 31% of patients were discharged from hospital. In addition, several studies [81,104,105] have reported the effective use of KALETRA in suppressing the activity of SARS-CoV-2. Medical teams in China [13], Singapore [99], Malaysia [100], and Thailand [97] have observed promising effects of KALETRA in combination with other medical interventions to treat COVID-19 patients, leading to many being discharged from hospitals. That being said, at present there is no available vaccine and medical treatment that could prevent COVID-19 infection. In addition, continuous on-going efforts are needed, both theoretical and clinical, to fully understand SARS-CoV-2 and COVID-19 for more effective treatment and prevention. 

## 7. Conclusions

The recent SARS-CoV-2 epidemic outbreak is an ongoing crisis that is causing global uncertainty on an unprecedented scale. The zoonotic pathogen, identified as an enveloped, positive-sense, single-stranded RNA beta-coronavirus with 96.2% similarity to that of a bat coronavirus (termed BatCoV RaTG13), is believed to have originated from infected bats and is transmissible between animals and humans, as well as between humans and humans, with up to 24 days of incubation period. By February 13, 2020, there were a staggering 60,412 confirmed cases of COVID-19, the disease associated with SARS-CoV-2 infection, and 1370 fatalities reported worldwide. Although the current mortality rate of COVID-19 is relatively low at approximately 2% compared to SARS (9.6%) and MERS (35%), the SARS-CoV-2 is highly contagious and has infected more patients than both SARS and MERS combined over a shorter period of time. A significant amount of collective work has been done in China and globally in the first 75 days of the outbreak, particularly in understanding the novel coronavirus, as well as in devising prevention and treatment strategies. Strict containment measures have been effectively implemented throughout China, particularly in infected areas, preventing uncontrolled spreading. Statistical studies have shown that the reproduction number (once as high as 5.7) has been on a declining trend, indicating lower spreading rates through containment and strict isolation measures. Promising medical interventions have been administered to COVID-19 patients with a total of 6271 patients discharged from hospitals in various countries (as of February 13, 2020). That being said, there are no available vaccines against the SARS-CoV-2 and little is still known about the post-effects of the COVID-19. Clearly, continued effort from across the world will be needed to fully understand the coronavirus family in order to prevent further and future outbreaks.

## Figures and Tables

**Table 1:** Recommendations by various governments and agencies. It should be noted that these are only selected recommendations, more information could be obtained at the respective references.

Nations/Organizations	Epidemic Prevention and Control
*Recommendations by various countries*	
China [60]	Keep physically clean, wash hands with clean soap or 75% alcohol solution for 20 seconds. Keep the room ventilated and sanitized. Frequently rinse contacted surfaces with 75% alcohol solution. Keep daily physical check-ups and seek for medical help if unwell. Wear mask/N95 mask. Avoid social activities, stay away from crowded areas, and keep distances from patients or suspected patients.
Canada [61]	Avoid contact with individuals with chronic conditions, compromised immune systems, and older adults. Limit taking public transit. Cover mouth and nose when coughing or sneezing. Use alcohol-based sanitizer if soap and water are not available. Avoid touching eyes, nose, or mouth with unwashed hands. Avoid non-essential travel to China. Avoid consuming uncooked food. Avoid contacting with animals or high-risk areas such as farms.
United Kingdom [62]	Cover nose and mouth with a disposable tissue when sneezing, coughing, wiping, and blowing the nose. Dispose used tissues into a waste bin. Stay indoors if travelled to mainland China, Thailand, Japan, Republic of Korea, Hong Kong, Taiwan, Singapore, Malaysia, or Macau in the last 14 days and if experiencing cough or fever or shortness of breath. Dispose clinical waste in leak-proof clinical waste bags or bins.
Australia [63]	Recommended to not travel to all of mainland China. Using a tissue and covering mouth when coughing or sneezing. Home isolation for 14 days if travelled from Hubei Province; left, or transited through, mainland China on or after February 1, 2020; or been in close contact with a confirmed case of COVID-19/.
Singapore [64]	Avoid eating raw or undercook meat. Avoid close contact with live animals.
*Recommendations by various agencies*	
Chinese Center for Disease Control and Prevention (CDC) [65]	Use an alcohol-based hand sanitizer with at least 60% alcohol if soap and water not readily available. Avoid touching eyes, nose, and mouth with unwashed hands. Cover cough or sneeze with a tissue, then throw the tissue in the trash. Avoid all nonessential travel to China. Get vaccinated and take anti-malarial drugs before travelling. Eat and drink safely. Keep away from animals and prevent bug bites. Avoid sharing body fluids.
MedlinePlus by the United States National Library of Medicine [66]	Avoid touching face, nose, or mouth with unwashed hands. Cover coughs and sneezes with a tissue. Then throw away the tissue and wash hands.

## References

[1] Nicks B.A. (2020). 2019 Novel Coronavirus (2019-nCoV): What We Currently Know.

[2] Phan T. (2020). Novel Coronavirus: From Discovery to Clinical Diagnostics.

[3] Lu R., Zhao X., Li J., Niu P., Yang B., Wu H., Wang W., Song H., Huang B., Zhu N. (2020). Genomic characterisation and epidemiology of 2019 novel coronavirus: Implications for virus origins and receptor binding. Lancet.

[4] Letko M., Marzi A., Munster V. (2020). Functional assessment of cell entry and receptor usage for SARS-CoV-2 and other lineage B betacoronaviruses. Nat. Microbiol..

[5] Zhu N., Zhang D., Wang W., Li X., Yang B., Song J., Zhao X., Huang B., Shi W., Lu R. (2020). A Novel Coronavirus from Patients with Pneumonia in China, 2019. N. Engl. J. Med..

[6] Fung T.S., Liu D.X. (2019). Human Coronavirus: Host-Pathogen Interaction. Annu. Rev. Microbiol..

[7] Gorbalenya A.E., Barker S.C., Baric S.R., de Groot J.R., Drosten C., Gulyaeva A.A., Haagmans B.L., Lauber C., Leontovich A.M., Neuman B.W. (2020). Severe acute respiratory syndrome-related coronavirus: The species and its viruses—A statement of the Coronavirus Study Group. bioRxiv.

[8] Peiris J.S.M., Lai S.T., Poon L.L.M., Guan Y., Yam L.Y.C., Lim W., Nicholls J., Yee W.K.S., Yan W.W., Cheung M.T. (2003). Coronavirus as a possible cause of severe acute respiratory syndrome. Lancet.

[9] Li W. (2005). Bats Are Natural Reservoirs of SARS-Like Coronaviruses. Science.

[10] Wang L.-F., Shi Z., Zhang S., Field H., Daszak P., Eaton B.T. (2006). Review of Bats and SARS. Emerg. Infect. Dis..

[11] De Groot R.J., Baker S.C., Baric R.S., Brown C.S., Drosten C., Enjuanes L., Fouchier R.A.M., Galiano M., E Gorbalenya A., Memish Z.A. (2013). Middle East Respiratory Syndrome Coronavirus (MERS-CoV): Announcement of the Coronavirus Study Group. J. Virol..

[12] World Health Organization (2020). World Health Organization. Middle East respiratory syndrome coronavirus (MERS-CoV). https://www.who.int/news-room/fact-sheets/detail/middle-east-respiratory-syndrome-coronavirus-(mers-cov).

[13] Chen N., Zhou M., Dong X., Qu J., Gong F., Han Y., Qiu Y., Wang J., Liu Y., Wei Y. (2020). Epidemiological and clinical characteristics of 99 cases of 2019 novel coronavirus pneumonia in Wuhan, China: A descriptive study. Lancet.

[14] Zhou P., Yang X.-L., Wang X.-G., Hu B., Zhang L., Zhang W., Si H.-R., Zhu Y., Li B., Huang C.-L. (2020). A pneumonia outbreak associated with a new coronavirus of probable bat origin. Nature.

[15] Mahase E. (2020). China coronavirus: What do we know so far?. BMJ.

[16] Jiang S., Du L., Shi Z. (2020). An emerging coronavirus causing pneumonia outbreak in Wuhan, China: Calling for developing therapeutic and prophylactic strategies. Emerg. Microbes Infect..

[17] Dong N., Yang X., Ye L., Chen K., Chan E., Yang M., Chen S. (2020). Genomic and protein structure modelling analysis depicts the origin and infectivity of 2019-nCoV, a new coronavirus which caused a pneumonia outbreak in Wuhan, China. bioRxiv.

[18] Zhao Y., Zhao Z., Wang Y., Zhou Y., Ma Y., Zuo W. (2020). Single-cell RNA expression profiling of ACE2, the putative receptor of Wuhan 2019-nCov. bioRxiv.

[19] Rothe C., Schunk M., Sothmann P., Bretzel G., Froeschl G., Wallrauch C., Zimmer T., Thiel V., Janke C., Guggemos W. (2020). Transmission of 2019-nCoV Infection from an Asymptomatic Contact in Germany. N. Engl. J. Med..

[20] Chan J.F.-W., Yuan S., Kok K.-H., To K.K.-W., Chu H., Yang J., Xing F., Liu J., Yip C.C.-Y., Poon R.W.-S. (2020). A familial cluster of pneumonia associated with the 2019 novel coronavirus indicating person-to-person transmission: A study of a family cluster. Lancet.

[21] Phan L.T., Nguyen T.V., Luong Q.C., Nguyen T.V., Nguyen H.T., Le H.Q., Nguyen T.T., Cao T.M., Pham Q.D. (2020). Importation and Human-to-Human Transmission of a Novel Coronavirus in Vietnam. N. Engl. J. Med..

[22] Wu J.T., Leung K., Leung G.M. (2020). Nowcasting and forecasting the potential domestic and international spread of the 2019-nCoV outbreak originating in Wuhan, China: A modelling study. Lancet.

[23] Li Q., Guan X., Wu P., Wang X., Zhou L., Tong Y., Ren R., Leung K.S., Lau E.H., Wong J.Y. (2020). Early Transmission Dynamics in Wuhan, China, of Novel Coronavirus–Infected Pneumonia. N. Engl. J. Med..

[24] Jaillon S., Berthenet K., Garlanda C. (2017). Sexual Dimorphism in Innate Immunity. Clin. Rev. Allergy Immunol..

[25] National Health Commission of the People’s Republic of China COVID-19 Outbreak Prevention, Control and Notification. http://www.nhc.gov.cn/xcs/yqtb/list_gzbd.shtml.

[26] World Health Organization Coronavirus Disease (COVID-2019) Situation Reports. https://www.who.int/emergencies/diseases/novel-coronavirus-2019/situation-reports.

[27] Roser M., Ritchie H., Ortiz-Ospina E. Coronavirus Disease (COVID-19)—Statistics and Research. https://ourworldindata.org/coronavirus.

[28] Zhao S., Musa S.S., Lin Q., Ran J., Yang G., Wang W., Lou Y., Yang L., Gao D., He D. (2020). Estimating the Unreported Number of Novel Coronavirus (2019-nCoV) Cases in China in the First Half of January 2020: A Data-Driven Modelling Analysis of the Early Outbreak. J. Clin. Med..

[29] World Health Organization Regional Office for Europe 2019-nCoV Outbreak is an Emergency of International Concern. http://www.euro.who.int/en/health-topics/health-emergencies/international-health-regulations/news/news/2020/2/2019-ncov-outbreak-is-an-emergency-of-international-concern.

[30] Guan W.-J., Ni Z.-Y., Hu Y., Liang W.-H., Ou C.-Q., He J., Liu L., Shan H., Lei C.-L., Hui D.S. (2020). Clinical characteristics of 2019 novel coronavirus infection in China. medRxiv.

[31] Huang C., Wang Y., Li X., Ren L., Zhao J., Hu Y., Zhang L., Fan G., Xu J., Gu X. (2020). Clinical features of patients infected with 2019 novel coronavirus in Wuhan, China. Lancet.

[32] Otter J., Donskey C., Yezli S., Douthwaite S., Goldenberg S., Weber D. (2016). Transmission of SARS and MERS coronaviruses and influenza virus in healthcare settings: The possible role of dry surface contamination. J. Hosp. Infect..

[33] Lei J., Li J., Li X., Qi X. (2020). CT Imaging of the 2019 Novel Coronavirus (2019-nCoV) Pneumonia. Radiology.

[34] Chung M., Bernheim A., Mei X., Zhang N., Huang M., Zeng X., Cui J., Xu W., Yang Y., Fayad A.Z. (2020). CT Imaging Features of 2019 Novel Coronavirus (2019-nCoV). Radiology.

[35] Lei H., Li Y., Xiao S., Lin C.-H., Wei D., Hu Z., Ji S., Norris S.L. (2018). Routes of transmission of influenza A H1N1, SARS CoV, and norovirus in air cabin: Comparative analyses. Indoor Air.

[36] Zumla A., Hui D.S., Perlman S. (2015). Middle East respiratory syndrome. Lancet.

[37] Chan J.F.-W., Kok K.-H., Zhu Z., Chu H., To K.K.-W., Yuan S., Yuen K.-Y. (2020). Genomic characterization of the 2019 novel human-pathogenic coronavirus isolated from a patient with atypical pneumonia after visiting Wuhan. Emerg. Microbes Infect..

[38] Cohen J. (2020). WHO Panel Puts off Decision on Whether to Sound Alarm on Rapid Spread of New Virus.

[39] Kupferschmidt K. (2020). Outbreak of Virus from China Declared Global Emergency.

[40] Kupferschmidt K. (2020). Study Claiming New Coronavirus can be Transmitted by People Without Symptoms was Flawed.

[41] Lanese N. How Far Could the New Coronavirus Spread?. https://www.livescience.com/how-far-will-coronavirus-spread.html.

[42] Imai N. (2020). Report 3: Transmissibility of 2019-nCoV 2020.

[43] Shen M., Peng Z., Xiao Y., Zhang L. (2020). Modelling the epidemic trend of the 2019 novel coronavirus outbreak in China. bioRxiv.

[44] Li X., Zai J., Wang X., Li Y. (2020). Potential of large “first generation” human-to-human transmission of 2019-nCoV. J. Med Virol..

[45] World Health Organization Novel Coronavirus (2019-nCoV) Advice for the Public. https://www.who.int/emergencies/diseases/novel-coronavirus-2019/advice-for-public.

[46] Riou J., Althaus C.L. (2020). Pattern of early human-to-human transmission of Wuhan 2019-nCoV. bioRxiv.

[47] Kucharski A.J., Russell T.W., Diamond C., Liu Y., Edmunds J., Funk S., Eggo R.M. (2020). CMMID nCoV working group Early dynamics of transmission and control of COVID-19: A mathematical modelling study. medRxiv.

[48] Ai L. (2020). Modelling the epidemic trend of the 2019-nCOV outbreak in Hubei Province, China. medRxiv.

[49] Zhao Q., Chen Y., Small D.S. (2020). Analysis of the epidemic growth of the early 2019-nCoV outbreak using internationally confirmed cases. medRxiv.

[50] Cao Z., Zhang Q., Lu X., Pfeiffer D., Wang L., Song H., Pei T., Jia Z., Zeng D.D. (2020). Incorporating Human Movement Data to Improve Epidemiological Estimates for 2019-nCoV. medRxiv.

[51] Bolker M.B., Champredon D., Earn D.J.D., Li M., Weitz S.J., Grenfell T.B., Dushoff J. (2020). Reconciling early-outbreak estimates of the basic reproductive number and its uncertainty: A new framework and applications to the novel coronavirus (2019-nCoV) outbreak. medRxiv.

[52] Huang Q., Herrmann A. (2020). Fast assessment of human receptor-binding capability of 2019 novel coronavirus (2019-nCoV). bioRxiv.

[53] Chen T., Rui J., Wang Q., Zhao Z., Cui J.-A., Yin L. (2020). A mathematical model for simulating the transmission of Wuhan novel Coronavirus. bioRxiv.

[54] Harvard University (2007). The Handiwork of Good Health.

[55] World Health Organization (2020). Novel Coronavirus (2019-nCoV) Advice for the Public.

[56] McDonnell G., Russell A.D. (1999). Antiseptics and Disinfectants: Activity, Action, and Resistance. Clin. Microbiol. Rev..

[57] Ellerin T. (2020). The New Coronavirus: What We do and don’t Know.

[58] U.S. Food & Drug Administration (2020). Masks and N95 Respirators.

[59] Gajanan M. (2020). Can Face Masks Prevent Coronavirus? Experts Say That Depends.

[60] National Health Commission of the People’s Republic of China Latest Tips to Prevent Novel Coronavirus. http://en.nhc.gov.cn/2020-02/05/c_76211.htm.

[61] Government of Canada 2019 Novel Coronavirus: Prevention and Risks. https://www.canada.ca/en/public-health/services/diseases/2019-novel-coronavirus-infection/prevention-risks.html#re.

[62] Public Health England Novel Coronavirus (2019-nCoV) Infection Prevention and Control Guidance. https://www.gov.uk/government/publications/wuhan-novel-coronavirus-infection-prevention-and-control/wuhan-novel-coronavirus-wn-cov-infection-prevention-and-control-guidance.

[63] Australia Government Department of Health Novel Coronavirus (2019-nCoV). https://www.health.gov.au/health-topics/novel-coronavirus-2019-ncov.

[64] Government of Singapore What You Can do to Protect Yourself from the 2019 Novel Coronavirus. https://www.gov.sg/article/what-can-you-do-to-protect-yourself-from-2019-ncov.

[65] Montvida O., Dibato J.E., Paul S. (2019). Evaluating the Representativeness of US Centricity Electronic Medical Records with Reports from Centers for Disease Control and Prevention: Demographics and Cardiometabolic Conditions. JMIR Med. Inf..

[66] MedlinePlus (2020). Coronavirus Infections. https://medlineplus.gov/coronavirusinfections.html#cat_79.

[67] Richman D.D., Nathanson N., Katze M.G. (2016). Chapter 20—Antiviral Therapy. Viral Pathogenesis.

[68] AHFS Patient Medication Information Oseltamivir. https://www.ahfsdruginformation.com/ahfs-patient-medication-information/.

[69] LV G. (2000). Influenza Virus Neuraminidase Inhibitors.

[70] AHFS Patient Medication Information (2016). Ganciclovir.

[71] Wishart D.S., Feunang Y.D., Guo A.C., Lo E.J., Marcu A., Grant J.R., Sajed T., Johnson D., Li C., Sayeeda Z. (2017). DrugBank 5.0: A major update to the DrugBank database for 2018. Nucleic Acids Res..

[72] Paules C.I., Marston H.D., Fauci A.S. (2020). Coronavirus Infections—More Than Just the Common Cold. JAMA.

[73] National Institute of Allergy and Infectious Diseases Developing Therapeutics and Vaccines for Coronaviruses. https://www.niaid.nih.gov/diseases-conditions/coronaviruses-therapeutics-vaccines.

[74] Sheahan T.P., Sims A.C., Leist S.R., Schäfer A., Won J., Brown A.J., Montgomery S.A., Hogg A., Babusis D., Clarke M.O. (2020). Comparative therapeutic efficacy of remdesivir and combination lopinavir, ritonavir, and interferon beta against MERS-CoV. Nat. Commun..

[75] World Health Organization WHO R&D Blueprint—Ad-hoc Expert Consultation on Clinical Trials for Ebola Therapeutics. https://www.who.int/ebola/drc-2018/treatments-approved-for-compassionate-use-update/en/.

[76] Ogbru O. Lopinavir and Ritonavir. https://www.medicinenet.com/lopinavir_and_ritonavir/article.htm#what_is_lopinavir_and_ritonavir_and_how_does_it_work_mechanism_of_action.

[77] US Food and Drug Administration (2000). Kaletra.

[78] Wang M., Cao R., Zhang L., Yang X., Liu J., Xu M., Shi Z., Hu Z., Zhou X., Xiao G. (2020). Remdesivir and chloroquine effectively inhibit the recently emerged novel coronavirus (2019-nCoV) in vitro. Cell Res..

[79] Richardson P., Griffin I., Tucker C., Smith D., Oechsle O., Phelan A., Stebbing J. (2020). Baricitinib as potential treatment for 2019-nCoV acute respiratory disease. Lancet.

[80] Li Y., Zhang J., Wang N., Li H., Shi Y., Guo G., Liu K., Zeng H., Zou Q. (2020). Therapeutic Drugs Targeting 2019-nCoV Main Protease by High-Throughput Screening. bioRxiv.

[81] Liu X., Wang X.-J. (2020). Potential inhibitors for 2019-nCoV coronavirus M protease from clinically approved medicines. bioRxiv.

[82] Tare D.S., Kode S.S., Hurt A.C., Pawar S.D. (2019). Assessing the susceptibility of highly pathogenic avian influenza H5N1 viruses to oseltamivir using embryonated chicken eggs. Indian J. Med Res..

[83] Werth B.J. (2018). Cephalosporins.

[84] Shahbaz K. (2017). Cephalosporins: Pharmacology and chemistry. Pharm. Boil. Evaluations.

[85] Papp-Wallace K.M., Endimiani A., Taracila M.A., Bonomo R.A. (2011). Carbapenems: Past, Present, and Future. Antimicrob. Agents Chemother..

[86] Aldred K.J., Kerns R.J., Osheroff N. (2014). Mechanism of Quinolone Action and Resistance. Biochemistry.

[87] Lei C., Fu W., Qian K., Li T., Zhang S., Ding M., Hu S. (2020). Potent neutralization of 2019 novel coronavirus by recombinant ACE2-Ig 2020. bioRxiv.

[88] Cohen J. (2020). Scientists are moving at record speed to create new coronavirus vaccines—But they may come too late. Science.

[89] Pardi N., Hogan M., Porter F.W., Weissman D. (2018). mRNA vaccines—A new era in vaccinology. Nat. Rev. Drug Discov..

[90] Park A. (2020). Inside the Company That’s Hot-Wiring Vaccine Research in the Race to Combat the Coronavirus.

[91] Chi H., Zheng X., Wang X., Wang C., Wang H., Gai W., Perlman S., Yang S., Zhao J., Xia X. (2017). DNA vaccine encoding Middle East respiratory syndrome coronavirus S1 protein induces protective immune responses in mice. Vaccine.

[92] Mazumdar T. (2020). Coronavirus: Scientists Race to Develop a Vaccine.

[93] Horie S., Gonzalez H.E., Laffey J.G., Masterson C. (2018). Cell therapy in acute respiratory distress syndrome. J. Thorac. Dis..

[94] Zumla A., Hui D.S., I Azhar E., A Memish Z., Maeurer M. (2020). Reducing mortality from 2019-nCoV: Host-directed therapies should be an option. Lancet.

[95] Ramaiah A., Arumugaswami V. (2020). Insights into Cross-species Evolution of Novel Human Coronavirus 2019-nCoV and Defining Immune Determinants for Vaccine Development. bioRxiv.

[96] Ahmed S.F., Quadeer A.A., McKay M.R. (2020). Preliminary identification of potential vaccine targets for 2019-nCoV based on SARS-CoV immunological studies. bioRxiv.

[97] Agence France Presse (2020). Wuhan Virus: Thailand Sees Apparent Success with Flu and HIV Drug Cocktail.

[98] Berlinger J. (2020). February 3 Coronavirus News.

[99] Kurohi R. (2020). Coronavirus: First Patient in Singapore Discharged, Some Given Anti-HIV Drugs.

[100] Channel News Asia (2020). Four-Year-Old Coronavirus Patient in Malaysia Discharged from Hospital.

[101] Croft J., Parks B. Both Illinois Coronavirus Patients have been Discharged from the Hospital, in CNN News. https://edition.cnn.com/2020/02/07/us/illinois-coronavirus-patients-discharge/index.html.

[102] Ram S. https://says.com/my/news/a-40-year-old-wuhanese-man-who-had-become-critical-at-jb-hospital-has-now-recovered.

[103] Chen H., Guo J., Wang C., Luo F., Yu X., Zhang W., Li J., Zhao D., Xu D., Gong Q. (2020). Clinical characteristics and intrauterine vertical transmission potential of COVID-19 infection in nine pregnant women: A retrospective review of medical records. Lancet.

[104] Lu H. (2020). Drug treatment options for the 2019-new coronavirus (2019-nCoV). Biosci. Trends.

[105] Li G., De Clercq E. (2020). Therapeutic options for the 2019 novel coronavirus (2019-nCoV). Nat. Rev. Drug Discov..

